# Massive Extraluminal gastrointestinal stromal tumour with pelvic extension: case report and mini-review of the literature

**DOI:** 10.1093/omcr/omaf093

**Published:** 2025-07-14

**Authors:** Yasir Alshareefy, Ali Alshareefy

**Affiliations:** St James's Hospital Dublin, James's Street, Dublin 8, D08NHY1, Ireland; Medcare Dr. Saeed Al-Shaikh Gastroenterology and Obesity Centre, Sheikh Zayed Road, SMJ Building, PO Box 215565, Dubai, United Arab Emirates

**Keywords:** gastroenterology, oncology, general surgery

## Abstract

Large extraluminal gastrointestinal stromal tumours (GISTs) are rare, with varied presentations and patient profiles. This report discusses the case of a 22-year-old female presenting with a 4-month history of lower abdominal pain, weight loss, and recurrent urinary tract infections. Imaging revealed a large intra-abdominal mass (9.3 × 15.3 × 18.9 cm) originating from the stomach, extending into the pelvis, and compressing adjacent structures. During surgery, the mass was found to arise from the lesser curvature of the stomach and adhered to the transverse colon, with non-adherent extension into the pelvis and compression of adjacent organs. A gastric wedge resection and transverse colectomy were performed with aim of achieving a R0 resection. Histopathological analysis confirmed a GIST and positive resection margins. A plan for adjuvant imatinib was initiated in accordance with the European Society for Medical Oncology (ESMO) guidelines. This article details the challenges faced in the management of such a rare presentation and highlights similar reports.

## Introduction

Gastrointestinal stromal tumours (GIST) are rare non-epithelial neoplasms, arising from the interstitial cells of Cajal and account for ~ 1% of all primary gastrointestinal tract (GIT) tumours with an annual incidence of 10–15 per million people [1]. The mean age of diagnosis of GIST is 50.6 years and there is an equal incidence between males and females, with the most common location being the stomach followed by small bowel and colon [2]. In this report we describe the case of a 22-year-old female who was found to have a large extra-luminal GIST originating from the lesser curvature of the stomach and transverse colon with non-adherent extension into the pelvis abutting the bladder. We also aim to explore the current literature regarding extra-luminal GIST and educate readers on the various challenges such a case poses to clinicians.

### Case

A 22-year-old female attended the gastroenterology outpatient clinic complaining of a 4-month history of lower abdominal pain, described as dull and heavy in nature and is exacerbated when working or walking for some distances, which has worsened in the last 2 weeks. In addition to this pain, the patient also reported a sensation of persistent bloating and infra-umbilical abdominal enlargement. She also experienced 5 kg unintentional weight loss over the last 3 months with altered bowel habits and recurrent urinary tract infections over the same period. Her background family history was unremarkable and with regards to lifestyle factors she did not smoke cigarettes and had very occasional alcohol consumption. Although it was not reported in the history, cross sectional imaging revealed previous gastric surgery with surgical staples, likely bariatric surgery.

On physical examination she appeared thin and pale with a lower abdominal prominence and everted umbilicus. On abdominal palpation a large mass was noted with the upper margin located just below the xiphisternum and the lower margin extending down to the pelvis. The overlying skin was normal, and no bruit could be heard over the mass.

Initial investigations included blood tests and ultrasound imaging of the abdomen and pelvis. Blood results including C-reactive protein, full blood count, renal and liver profiles and tumour markers such as CA-125 were unremarkable. Ultrasound examination demonstrated a well-circumscribed echogenic solid lesion in the middle of the pelvis abutting the urinary bladder and uterus with no obvious free collection or lymphadenopathy. The initial impression based off of these investigations was a tumour originating in the pelvis of possible gynaecological origin.

**Figure 1:**
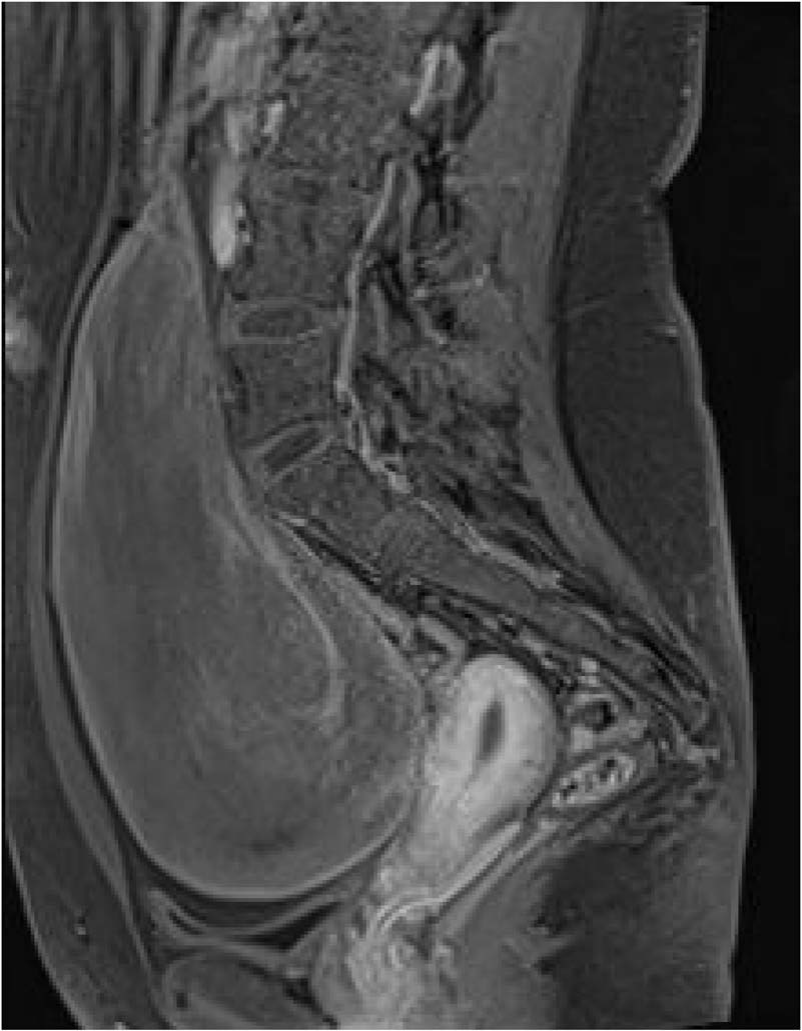
MRI abdomen and pelvis with contrast (sagittal view) demonstrating a 9.3 × 15.3 × 18.9 cm well defined, solid space occupying lesion displacing the urinary bladder inferiorly and the uterus and ovaries into the pre-sacral area.

**Figure 2:**
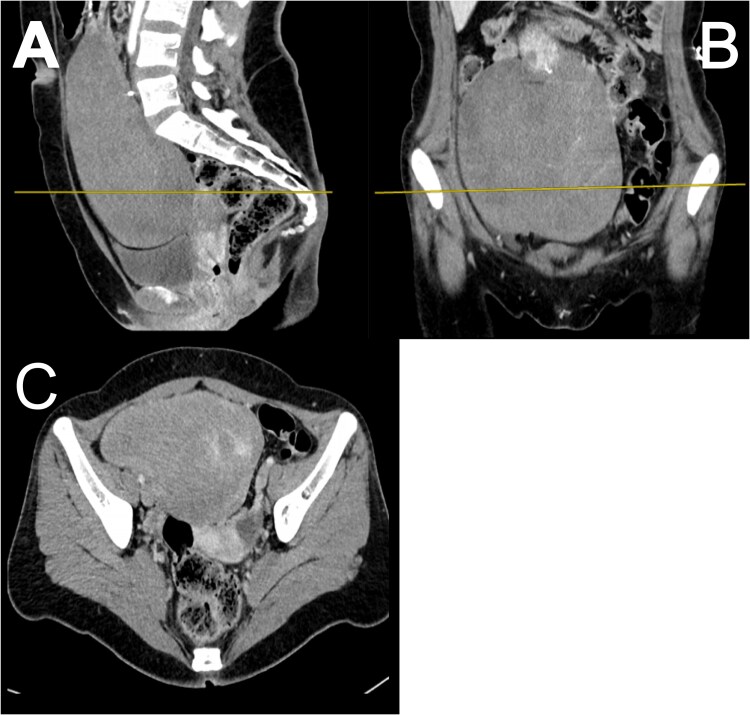
Sagittal (A), coronal (B) and axial (C) CT abdomen and pelvis showing the large mass and likely point of origin from the anteroinferior aspect of the gastric remnant, no evidence of metastasis.

These findings were explained to the patient and a plan was agreed upon to further investigate and characterise this abdomino-pelvic mass. Contrast enhanced magnetic resonance imaging (MRI) of the abdoman and pelvis was performed and revealed a 9.3 × 15.3 × 18.9 cm well defined predominantly solid heterogeneously enhancing abdomino-pelvic lesion with imaging characteristics, location and relationship with adjacent structures suspicious for a mesenteric desmoid tumour (Fig. 1). Subsequent Computed Tomography (CT) scan of the thorax, abdomen and pelvis confirmed the described mass with no radiological evidence of distant metastases or lymphadenopathy and raised suspicion for an exophytic GIST as the lesion is inseparable from the anteroinferior wall of the gastric remnant (Fig. 2).

A multi-disciplinary meeting including a gastroenterologist and general surgeon was arranged and a plan exploratory laparotomy +/− resection of the tumour was discussed and agreed upon by the patient.

**Figure 3:**
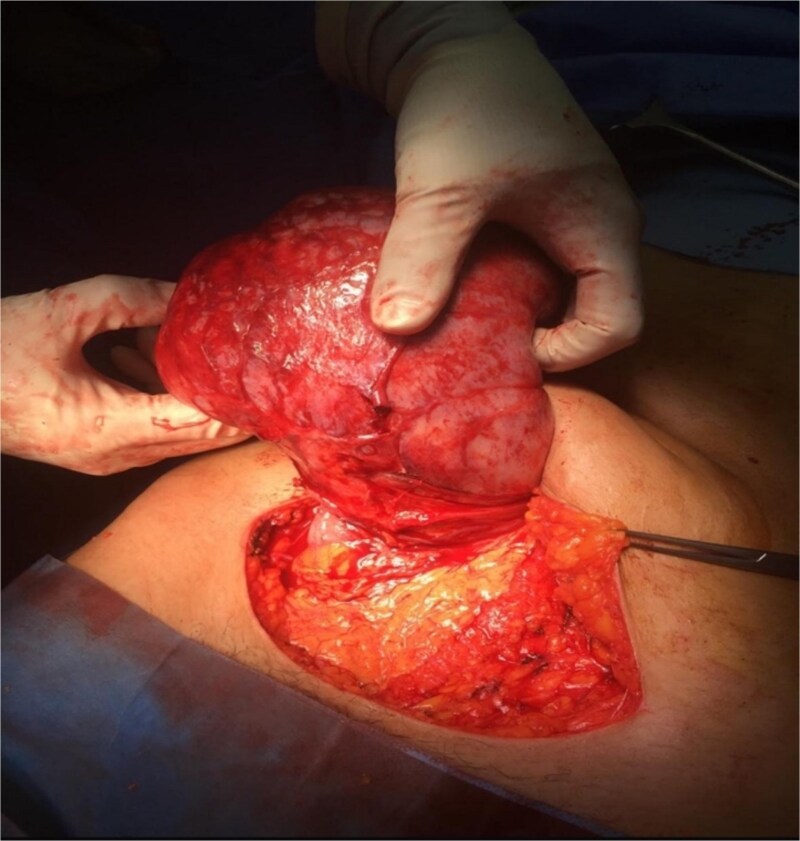
Gross surgical specimen.

**Figure 4:**
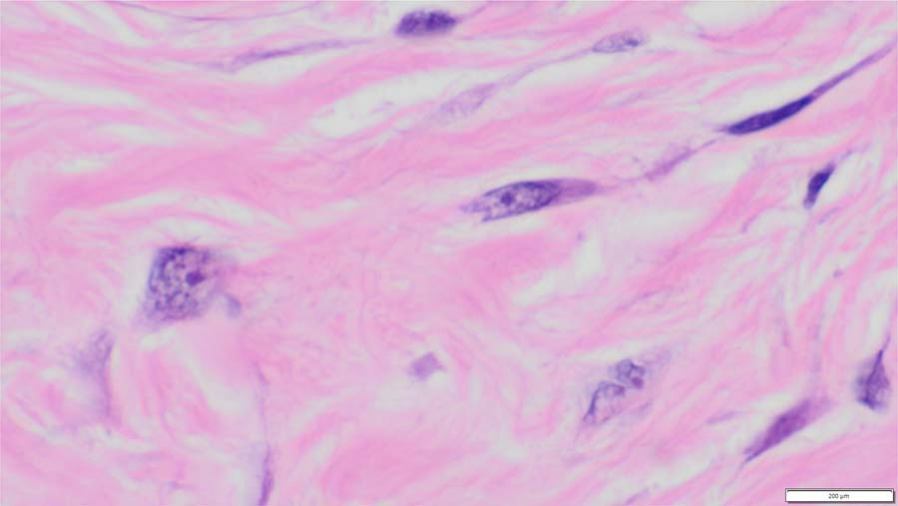
×1000 magnification showing spindle cells with hyperchromatic pointed end bland nuclei and some cells with oval to plump granular chromatin nuclei and inconspicuous nucleoli, elongated eosinophilic cytoplasm and thin collagen bands.

**Figure 5:**
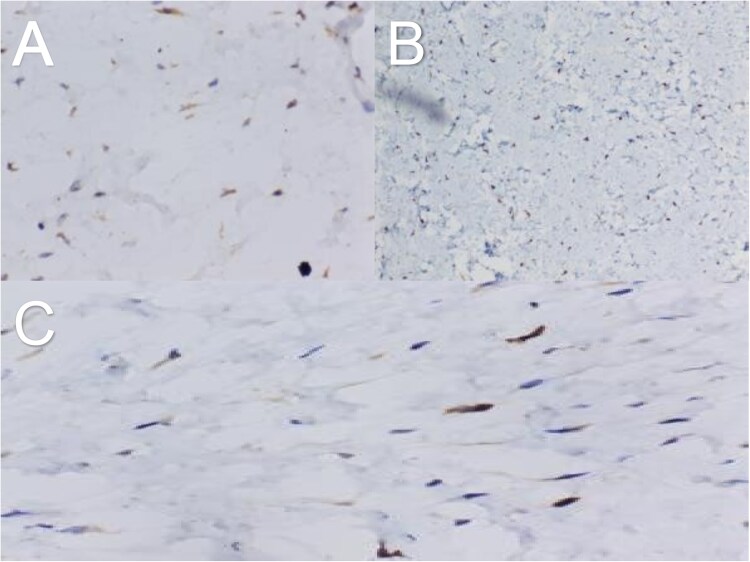
Immunohistochemistry showing tumour cells positive for SDHB (A), CD117 (B) and DOG-1 (C).

Under general anaesthesia, aseptic conditions and lying in the supine position incision was made in the skin and dissected down to the peritoneum, which was subsequently extended superiorly, revealing a large mass which appears to have originated from the lesser curvature of the stomach and transverse colon and extended down into the pelvis with no attachment to intra-pelvic organs. An intra-operative gastroscopy was performed and showed gastric indentation at the antrum and a collapsed and deformed cavity, however there was no mucosal abnormality. Fine dissection of the mass and separation of the mass from the gastric lesser curvature proved difficult, thus a 5 cm wedge section of the stomach was resected and repaired with stapling and suturing, alongside a 3 cm section of the transverse colon, which was repaired with an end-to-end anastomosis, with the purpose of achieving a R0 resection. After full haemostasis was achieved the abdomen was closed in layers. The surgical specimen (Fig. 3) was sent for histopathological analysis (Fig. 4) which demonstrated a tumour consistent with GIST of spindle type, absence of necrosis and lymph nodes in the mass. Immunohistochemistry staining of the tumour cells were positive for c-KIT, DOG-1, Smooth Muscle Actin, CD-117 and SDH-B (Fig. 5). The cells were negative for S-100, CD-34, Beta-catenin and caldesmon. The Ki-67 index was 1%. The resection margin was positive for tumour cells.

The post-operative period was uneventful, the patient was kept nil by mouth on total parenteral nutrition for 1 week prior to returning to a graduated soft oral diet and was discharged home with an outpatient review in 2 weeks. Currently at the time of writing this report the patient has been referred to oncology and initiated on imatinib 400 mg per day for a provisional duration of 1 year and at 6-month review there was no clinical or radiological recurrence noted. The decision for adjuvant imatinib therapy was made due to the large size of the tumour deeming it to be high risk according to the NIH-Fletcher criteria [3]. European Society for Medical Oncology (ESMO) guidelines states for high risk tumours adjuvant therapy with imatinib for 3 years is recommended [4]. It is important to note the ESMO guidelines do not recommend positive margins as a means of assessing for need of adjuvant therapy thus the primary factor in this decision rested with the tumour size [4]. A provisional plan for 1 year of imatinib therapy to assess tolerance was put in place with view to extend to 3 years in accordance with ESMO guidelines [4].

### Literature review

A literature search was conducted on PubMed using the keywords ‘extraluminal,’ ‘gastrointestinal stromal tumours,’ and ‘GIST.’ Boolean operators were applied to refine the search strategy. Only articles published in English were included. Publications were screened for relevance based on title and abstract, with full-text reviews conducted for eligible studies. Case reports, case series, and clinical studies discussing large extraluminal GISTs were considered.

Three reports noted a rare presentation of an extraluminal jejunal GIST presenting with a life-threatening bleed/hemoperitoneum [5–7]. The symptoms varied drastically between these 3 cases with loss of consciousness, a 3-day history of melena, haematochezia and colicky abdominal pain and vague abdominal pain. This highlights the complexity of this condition and the varying presentations despite a common diagnosis. The age of these patients ranged from 46–87 with a mean of 62.3 and 2/3 patients mentioned in these reports were of male sex. Each case was managed effectively with laparotomy and excision of the bleeding tumour and in 2 cases [5, 6] post-operative adjuvant imatinib therapy was initiated. In one case, surgical therapy alone was sufficient and the patient was followed up with no evidence of recurrence at 16 months [7]. The size of the tumours varied from 4-13 cm × 3.5-11 cm.

Another report [8] describes the case of an 86-year-old male presenting initially with anorexia, fatigue, intermittent vague abdominal pain and 10 kg weight loss over the past 6 months, followed by lower urinary tract symptoms, namely frequency and mild dysuria. A CT scan was performed and revealed a 13 × 11 × 9 cm presacral lesion compressing the bladder anteriorly and on laparotomy was found to have a fistula tract from the ileal lumen. The lesion was excised and a 1-year course of adjuvant imatinib was planned and at the time of publication no recurrence was noted.

Wang and colleagues [9] published a similar case to ours with regards to an extraluminal GIST arising from the lesser curvature of the stomach. A sample was taken with endoscopic ultrasound guided fine needle aspiration and histopathology confirmed a GIST. The tumour which measured 10 × 8 × 8 cm was successfully excised laparoscopically with negative surgical margins. The patient was followed up at 6 months and 1 year and no recurrence was noted. Key differences in this case include the age of the patient being 62 and the presentation; this mass was noted incidentally during a routine health examination and ultrasonography of the abdomen.

We also found a report of a 34-year-old female who presented with a 4-month history of an epigastric mass [10]. Initial laboratory workup was unremarkable, and a contrast enhanced CT revealed a 8 × 7 cm mass in relation to the transverse colon. On laparotomy the mass was found to originate from the serosal aspect of the transverse colon and was adherent to the stomach, gallbladder and pancreas and was resected via transverse colectomy with end-to-end anastomosis and loop ileostomy formation and cholecystectomy as it could not be freed from the gallbladder. Again, this patient was not given adjuvant chemotherapy and was asymptomatic at 8 months post-op.

As we can see, large extraluminal GISTs are a particularly rare entity and various reports have been published with wide range of patient ages, presenting symptoms and tumour location. It is worth highlighting that all the reports of extraluminal GIST found in our literature search were found in non-Caucasian individuals with a predominance of the cases being in Asian populations. Regardless of the presenting symptoms, all reports included adopted a similar diagnostic approach; a CT abdomen was performed and detected the mass, followed by exploratory laparotomy and resection +/− adjuvant chemotherapy. A key difference in our case to those already published is the age of our patient and the size of the tumour however, a similar case of a huge abdomino-pelvic malignant GIST measuring 25.3 × 20 × 14 cm in a 42-year-old woman with neurofibromatosis type 1 (NF1) was published by Omar and colleagues [2]. The link between NF1 and GIST has long been known, however the genetic basis for the etiopathogenesis is not well understood.

### Challenges and recommendations

Extra-luminal GIST poses a significant dilemma with regards to surgical planning and management. Cross-sectional imaging techniques such as CT and MRI can help define the spatial relationship of large intra-abdominal lesions which can aid in the preoperative management of such cases, however we found that direct visualisation was superior as we were able to see the origin of the tumour from the lesser curvature of the stomach and the adherence to the transverse colon which imaging failed to detect. This finding is validated by the studies included in our literature review where a similar approach was taken. We used intraoperative gastroscopy which showed antral luminal indentation and no mucosal abnormality which helped confirm the origin of the tumour from the lesser curve, however in retrospect, an oral contrast enhanced CT could have been utilised and revealed any luminal abnormalities and show areas of luminal collapse where the tumour may be located, which would then have allowed for more accurate preoperative assessment of the lesion and aided in the surgical planning of the case. Intra-operatively, the point of origin from the lesser curve was identified and a wedge gastrectomy was performed to preserve as much of the organ as possible due to the significant complications of a partial/full gastrectomy and effect on quality of life considering the young age of our patient. Attempts were made to free the mass from the transverse colon, but we were unable to do so, thus a salvage transverse colectomy was performed with end-to-end anastomosis. We could not find any other cases with a similar operative approach due to the rarity of this condition, thus it is difficult to comment on optimal surgical approach and again highlights the difficulty extra-luminal GIST presents.

The role of endoscopy in the diagnosis of GIST has been well documented, particularly with intraluminal GIST and the detection of a submucosal lesion most commonly on oesophagogastroduodendoscopy (OGD), however endoscopy may also aid the diagnosis of extraluminal GIST. Endoscopic ultrasound guided fine needle aspiration (EUS-FNA) is becoming increasingly used in the diagnosis of gastrointestinal tumours, however there are concerns when it comes to sampling intra-peritoneal lesions due to the risk of seeding metastases. A case of an extraluminal duodenal GIST diagnosed with EUS-FNA [11] has been reported as being safe and effective, thus can be considered for use with extraluminal GIST.

## Conclusion

Extraluminal GIST remain a rare subtype of GIST with only a few reports available in the literature. We have highlighted the difficulties one faces when encountered with such a case, from the diagnosis to the management. Much more research is required to answer the various questions posed in our article so we can define the optimal management of such cases. We recommend researchers to continue to publish their findings with similar reports to continue to help educate readers and raise awareness of this topic.
